# Effects of a Natural Mordenite as Pozzolan Material in the Evolution of Mortar Settings

**DOI:** 10.3390/ma14185343

**Published:** 2021-09-16

**Authors:** Jorge L. Costafreda, Domingo A. Martín, Leticia Presa, José Luis Parra

**Affiliations:** 1 Escuela Técnica Superior de Ingenieros de Minas y Energía, Universidad Politécnica de Madrid, C/Ríos Rosas, 21, 28003 Madrid, Spain; domingoalfonso.martin@upm.es (D.A.M.); Leticia.presa.madrigal@alumnos.upm.es (L.P.); joseluis.parra@upm.es (J.L.P.); 2 Laboratorio Oficial para Ensayos de Materiales de Construcción—LOEMCO, Calle Eric Kandel 1, TecnoGetafe, 28906 Getafe, Spain

**Keywords:** cement, mordenite, mortars, pozzolan, mechanical strength

## Abstract

This paper shows the results of a study focused on the evolution and properties of mortars made with a mixture of portland cement (PC) and natural mordenite (Mor). To begin, samples of mordenite, cement and sand were studied with X-ray diffraction (XRD), X-ray fluorescence (XRF) and granulometric analysis (GA). Next, mortars with a ratio of 75% PC and 25% mordenite were prepared to determine their initial and final setting times, consistency and density. Continuing, the density, weight and compressive strength of the specimens were determined at 2, 7, 28, 90 and 365 days. Finally, the specimens were studied using SEM, XRD and XRF. The results of the study of the mordenite sample showed a complex constitution where the major mineral component is mordenite, and to a lesser degree smectite (montmorillonite), halloysite, illite, mica, quartz, plagioclase and feldspar, in addition to altered volcanic glass. Tests with fresh cement/mordenite mortar (CMM) showed an initial setting time of 320 min and a final setting time of 420 min, much longer than the 212–310 min of portland cement mortar (PCM). It was established that the consistency of the cement/mordenite mortar (CMM) was greater than that of the PCM. The results of the density study showed that the CMM has a lower density than the PCM. On the other hand, the density of cement/mordenite specimens (CMS) was lower than that of portland cement specimens (PCS). The CMS compressive strength studies showed a significant increase from 18.2 MPa, at 2 days, to 72 MPa, at 365 days, with better strength than PCS at 28 and 365 days, respectively. XRD, XRF and SEM studies conducted on CMS showed a good development of primary and secondary tobermorite, the latter formed at the expense of portlandite; also, ettringite developed normally. This work proves that the partial replacement of PC by mordenite does not have a negative effect on the increase in the mechanical strength of CMS. It indicates that the presence of mordenite inhibits the spontaneous hydration of C_3_A and controls the anomalous formation of ettringite (Ett). All this, together with the mechanical strength reported, indicates that mordenite has a deep and positive influence on the evolution of the mortar setting and is an efficient pozzolan, meaning it can be used in the manufacture of mortars and highly resistant pozzolanic cement, with low hydration heat, low density, stability in extremely aggressive places and a low impact on the environment.

## 1. Introduction

Currently, the study of new natural and artificial resources to improve the quality of cement, mortars and concretes has increased greatly, all these materials are grouped under the name of pozzolans [1]. Natural pozzolans are aluminosilicates that do not have their own cement properties that once grounded up react with calcium hydroxide when there is water present [2]. There are many natural pozzolans, and they come in many forms and types [3]. Natural pozzolans are found in many varied geological environments, which are related to pyroclastic deposits rich in glass, ignimbrites, obsidian, perlite, tuff, volcanic ash, shale, cherts, diatomites and slates [4]. It is common to pre-burn these materials to obtain the best pozzolanic performance in cement factories [5,6,7,8]. Artificial pozzolans are the result of industrial activities, such as blast furnace slag, fly ash, silica fume, remains of ground-up bricks and shingles, ash from rice husks and almonds [6,9,10,11]. The manufacture of eco-efficient concretes using artificial pozzolans is a common practice today [12]. In the last few decades, the use of zeolites as natural pozzolans has increased [13,14,15], mainly clinoptilolite and heulandite [16]. The use of zeolites is due to their abundance, low mining costs, large and varied deposits, and exceptional physical and chemical properties. Zeolites represent the most varied and abundant mineral group in the earth’s crust [17]. The main properties of zeolites are microporosity (about 50% of the intracrystalline space is empty), ionic transfer, Si/Al ratio, surface area, adsorption capacity, low density, thermal stability and neutral behaviour [18,19]. The mixture of zeolite with cement in the presence of water creates more reaction stable products, with hydraulic properties, such as portlandite and tobermorite [20]. These mineral phases are formed by a combination of other anhydrous phases present in the original chemical composition of portland cement. The mixture of zeolite exerts a marked influence on the reaction process and allows the formation of new products that favour the durability of cement, mortars and concretes against the sulphate attack [20,21,22,23]. Portland cement mixes with pozzolans have high mechanical strength, durability and low reactivity against alkalis [24]. Generally, these mixtures have been successfully used in the manufacture of seawater-resistant concretes, and in structures built in soils composed of calcium and magnesium sulphates. Pozzolanic cements are also used in foundation works in gypsiferous soils [25]. The use of pozzolans prevents thermoplastic retraction and cracking of mortars [26]. Pozzolanic cements are capable of developing abrasion resistance, cohesion, workability, impermeability, resistance to freeze–thaw cycles, humidity control and volume stability [27]. Nowadays, the extensive knowledge of the properties of natural zeolites depicts their advantage over other non-metallic natural raw materials, due to low extraction and processing costs, easy access to deposits, versatility and low environmental impact [28]. However, despite these obvious advantages, the properties of zeolites vary considerably from species to species [29]. Currently, only a few varieties of zeolites are used, among these are heulandite and clinoptilolite. Marvila et al. [30] proved that the use of açaí fibre improves the durability of mortars in aggressive natural conditions, such as salt mist attack and thermal shock. Sedaghatdoost et al. [31] researched the properties of mortars made with scrap glass powder as a substitute for cement in 0, 5, 10 and 15% for construction under high-temperature conditions, the results were an improvement of the mechanical strengths of mortars by 17% at high temperatures. The best result was obtained at 10%. Selvaranjan et al. [32], on the other hand, used rice husk ash as a substitute for the fine/filling aggregate material in the mortar at 0, 10, 20, 30 and 50%; 30% was the most effective proportion to maintain mechanical strength, improve thermal performance and reduce thermal conductivity by up to 62%. Xiong et al. [33] used ceramic polishing brick powder as a supplementary cementation material to replace fly ash in the cementitious compounds of engineering works. They showed that with a dosage of 35% the rate of resistant activity increased significantly from 28 days to 14.29%, exceeding the strength of fly ash. Shao et al. [34] have researched the mechanical and retraction behaviour of cement mortar with rubber particles from used tires and an expansive agent. They proved that both rubber particles and the bonded expansive agent reduce the risk of cracking, retraction and loss of mechanical strength. Barreto et al. [35] evaluated the residues of bricks made with kaolinite clays for reuse in structural concretes, due to their high pozzolanicity. They found that the variation in the electrical conductivity of this residue was 1.21 mS/cm. Furthermore, the addition of 10% yielded results of 27.2 MPa at 14 days and 31.42 MPa at 28 days, whereas with the addition of 20% the mechanical strength was 24.5 MPa at 14 days and 27.82 MPa at 28 days, respectively. Mordenite is also well known in the field of building materials, as reported in the works of Kitsopoulos and Dunham [36], Vigil de la Villa et al. [37], Mertens et al. [38]. The main objective of this work is to demonstrate experimentally the effects of a zeolitic pozzolan on the mortar setting, and its possible use in the industrial manufacture of mortars, concretes and pozzolanic cements. The results obtained can serve as a guide applicable to any type of pozzolan.

## 2. Materials and Methods

### 2.1. Materials

The materials used in this research consisted of natural mordenite (Mor) and common portland cement. The mordenite comes from the Collado de Las Hermanicas, in the Los Frailes caldera, located in the province of Almería, in south-eastern Spain. The outcrop is located at the following coordinates: 36°46′41″ N and 2°04′17″ W. This research used portland type I cement, CEM I 42.5 R (Standard UNE-EN 197-1:2011) [39]. The fine aggregate consisted of a standard sand (SS) called CEN-NORMSAND DIN EN 196-1. This test sand is used to determine the strength of the cement in accordance with DIN EN 196: 2016-11 [40], which establishes the following essential criteria for this test sand: natural rounded sand rich in quartz, granulometric distribution (determined by granulometric analysis), weight tolerances: 1345 and 1355 g ± 5 g, i.e., between 1345 and 1355 g per bag and maximum humidity <0.2%.

### 2.2. Methods

In the first stage, the materials used in this work were analysed by X-ray diffraction (LOEMCO, Madrid, Spain) (XRD) in their natural and anhydrous state. In the second stage of the research, hardened and cured specimen fragments were analysed at 7, 28 and 90 days. This analysis was performed by the crystalline powder method (PTE-RX-004). The equipment used was a PANalytical XPERT PRO MPD (LOEMCO, Madrid, Spain), with a copper tube (45 kV, 40 mA).

A Philips-505 scanning electron microscope (SEM) (LOEMCO, Madrid, Spain) with an EDAX 9000 was used. The samples were uncoated. First of all, the mordenite sample was analysed in its natural state and later the solidified and cured specimens were analysed at 7, 28, 90 and 365 days.

A Philips-PW 1404 was used for X-ray fluorescence (LOEMCO, Madrid, Spain) (XRF). Four types of samples were analysed: mordenite in its natural state, anhydrous portland cement, standard sand (SS) and PCS and CMS specimens. The samples were crushed up to 200 mesh, and then 8 g were weighed and mixed with elbaite (1.5 mL). Next, they were dried at room temperature for 5 min. Test pads were prepared of 5 cm each.

The loss of ignition was determined in an oxidising atmosphere (Standard UNE-EN 196-3:2005 [41]). The sample was calcined at a temperature of 950 °C, in order to remove CO_2_ and water. The test procedure was as follows: 1.0 g of sample was weighed and placed in a crucible. It then was placed in an electric furnace at a controlled temperature of 950 °C for 5 min. The sample was cooled in the desiccator to room temperature. Finally, the mass constancy (MC) was determined. The loss on ignition was calculated using the following Equation (1):

(1) $$\mathrm{LOI}=M_{\mathrm{ST}}-\frac{M_{\mathrm{CST}}}{M_{\mathrm{ST}}}\times 100$$

where: M_ST_: mass of the sample tested (g); M_CST_: Mass of the calcined sample tested (g).

The geometric reduction of the mordenite used in this research was carried out to determine both the granulometric distribution and the fine particle content, in accordance with Standard UNE-EN 933-1:2012 [42]. In the grinding and crushing phase, the following equipment was used: Alas crusher, Controls crusher, Siebtechnik vibrating mill and a Selecta stove (LOEMCO, Madrid, Spain). A sieve N° 26/13 (0.063–200 mm in diameter-AISI 304) was used in the sieving of the samples. A second sieving was carried out with a fraction of 0.063 mm fraction following the methodology stated in Standard UNE-EN 933-10:2010 [43]. Basically, the test was developed in three phases: the first phase yielded a 4 cm diameter, in the second phase the diameter was reduced to 1 cm, and in the third phase the sample was completely ground up. The material of the third phase underwent a granulometric analysis using the filler method [43]. Some of the material from the third grinding phase underwent a granulometric analysis with the Mastersizer 2000-Malvern laser granulometer (LOEMCO, Madrid, Spain), with a Hydro2000MU module, by which the BET surface and fine particle diameters were determined. The object of this test was to determine how much the diameter of the mordenite particles could be reduced, maintain this uniformity until obtaining Blaine granulometry of portland cement.

The granulometric analysis of standard sand (SS) was carried out following the Standard UNE-EN 933-1:2012 [42].

The water absorption capacity (W_AB_) of the sample was measured using a pycnometer, in accordance with UNE-EN 1097-6:2000 [44]. The sample was reduced and divided into two sub-samples (Mor-1 and Mor-2); each sample had a volume between 0.5 and 0.6 litres. They were then washed on a 4 mm sieve to remove the finer particles. The particles retained on the 31.5 mm sieve were discarded. Both samples were dried in an oven at 110 °C until a constant mass was obtained. The samples were cooled to room temperature. Then the two samples were placed in the pycnometer. Each water absorption value was calculated rounded to the nearest 0.1% fraction. The average value obtained from both sub-samples was used as the water absorption (W_AB_) at each measurement time. Water absorption was calculated using the following Equation (2):

(2) $$W_{\mathrm{AB}}=W_{F}-\frac{M_{F}-M_{I}}{m_{2}-m_{1}}\times 100$$

where W_F_: water absorption at the final moment of measurement, M_F_: mass of the pycnometer at the final measurement time (g), M_I_: mass of the pycnometer at the intermediate measurement times (g), m_2_: mass of the dry pycnometer and sub-samples (Mor-1 and Mor-2) tested (g), m_1_: mass of the pycnometer (g).

Standard UNE-EN 196-1:2018 [45] was used in the preparation and dosing of cement/mordenite (CMM) and portland cement (PCM) mortars. The ratio of materials in cement/mordenite mortars (CMM) was as follows: cement: 375 g, mordenite: 125 g, fine aggregate (SS): 1350 g and distilled water: 225 g. In portland cement mortars (PCM) the dosage was: PC: 450 g, SS: 1350 g and distilled water: 225 g; the object of the PCM was to monitor the behaviour of the CMM throughout the curing period. Portions of fresh CMM and PCM mortars were used for tests of initial and final setting times, as well as consistency and density. The other portions of the mortars were placed in standardised moulds for curing in the wet chamber for 2, 7, 28, 90 and 365 days, after which the specimens, with standard dimensions of 160 × 40 × 40 mm, were unmoulded and used for tests of density, weight, compressive strength, SEM, XRD and XRF. Table 1 summarises the procedure followed in the dosage of the mortars.

A Suzpecar-C-700 concrete mixer and Bonfiglio-MVF 44 compactor were used to make the mortars. Fresh mortars were placed in a humidity chamber at 20 °C ± 1 °C and 90% relative humidity for 24 h [45]. Mechanical strength tests were performed at 2, 7, 28, 90 and 365 days (Table 1).

In determining the initial and final times of the setting, as well as the stability of the volume, a Vicat apparatus and a Le Chatelier mould were used, according to Standard UNE-EN 196-3:2005 [41]. A cement mixture with mordenite was prepared in proportion 75:25% and 225 g of distilled water [45] UNE-EN 196-1:2018, which was subsequently placed in a wet chamber at 20 °C and relative humidity of 90%, for 24 h. In order to determine volume stability, a mixture of 500 g of portland cement, mordenite and distilled water was made and mixed for 10–15 s. The room temperature of the chamber was 19.1 °C, with relative humidity at 64%.

Consistency is a measure of fluidity and humidity of fresh mortar, which gives a measure of its deformability when subject to specific stress. The consistency was determined by the vibrating table, and the test procedure was based on the Standard UNE-EN 1015-3-2000 [46]. Meaning what was determined was the value of the horizontal scattering of the mortar mass in mm.

The horizontal scattering of the mortar mass was determined by measuring the diameter of the fresh mortar samples placed in a tapered mould of stainless steel 60 mm high, with an inner diameter of 100 mm at the base and 70 mm at the top. Once removed from the mould, the mortar was placed on a stainless-steel vibrating table made up of a frame, a rigid plate, a disc, a horizontal axis, a lifting cam and a lifting shaft. Next 15 vertical shakes were applied, lifting the shaking table, and letting it fall freely from a certain height; this caused the scattering of the mortar mass on the disc. The mass was measured twice, taking the mean value in mm of both measurements with an accuracy of 1 mm. The standard states that mortar densities >1200 kg/m^3^ the scattering must be equal to 185 mm. The greater the fluidity of a mortar, the lower its workability.

To determine the density of fresh mortars and specimens, a portion of fresh cement/mordenite mortar (FCMM) and fresh portland cement mortar (FPCM) with the known weight of (m_1_) was deposited in a container with an inner diameter of 125 mm and a 1 litre capacity (V_v_). Standard blows were applied to expel the bubbles and then the container was weighed (m_2_) according to Standard UNE-EN 1015-6:1999/A1:2007 [47].

The apparent density ($\rho_{\mathrm{FM}}$) of FCMM and FPCM was calculated using the following Equation (3):

(3) $$\rho_{\mathrm{FM}}=\frac{m_{1}-m_{2}}{V_{v}}$$

To determine the apparent density of the cement/mordenite specimens (CMS) and portland cement (PCS) fragments of these specimens were used that had previously been dried on a stove. They were then weighed (M_1_) and covered with a layer of paraffin; the weight (M_2_) was determined again. All the solidified specimens were waterproofed and submerged in a water container with a built-in scale, through which a third weight reading was obtained (M_3_).

The apparent density of the waterproofed specimens was calculated using the following Equation (4):

(4) $$\rho_{\mathrm{as}}=M_{2}/\left[M_{2}-\left(M_{3}/\rho_{3}\right)\right]$$

where ρ_as_: the apparent density of the waterproofed specimens, ρ_3_: the apparent density of the water (0.9998395 g/mL at 4.0° Celsius).

The compressive strength was carried out according to the specifications of Standard UNE-EN 196-1:2018 [45]. An SDE multi-testing machine, model MEM-101/20-SDC-5523, with a speed of charge of 2400 ± 200 *n*/s was used to determine the compressive strength. Mechanical strength determination was based on the following Equation (5):

(5) $$R_{c}=\frac{F_{c}}{1600}$$

where R_c_: Compression strength (Newton/mm^2^), F_c_: Maximum breakage load (Newton), 1600: product of 40 mm × 40 mm (plane surface in mm^2^).

## 3. Results

### 3.1. X-ray Diffraction (XRD)

**Figure 1:**
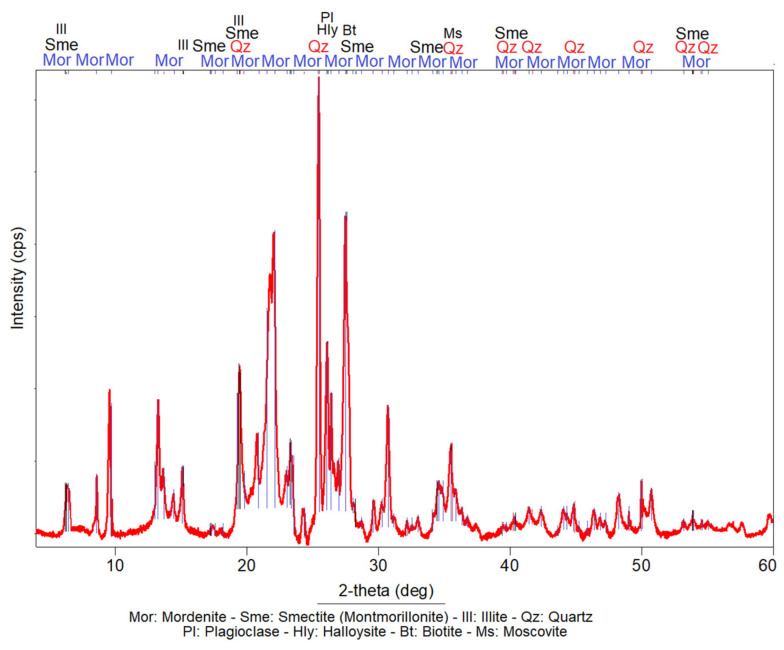
X-ray diffraction patterns obtained from the analysed sample.

According to the XRD analysis, the investigated sample is mainly composed of mordenite. The accompanied secondary phases are smectite (montmorillonite), illite, quartz, plagioclase, biotite, muscovite and halloysite (Figure 1). A maximum of 52 peaks of mordenite were detected. The higher intensity peaks (pk.) are located at the following positions of angle 2θ: pk.15 (25.45); pk.12 (21.95); pk.20 (27.49); pk.11 (20.82); pk.16 (26.09) and pk.9 (19.48). Treacy and Higgins [48] set the maximum intensity peak of mordenite at the angular position of 2θ: 6.51; according to these authors, the other peaks of greater intensity of this mineral are located at 2θ: 9.77, 13.45, 22.20, 26.25, 27.67, 27.87 and 25.63. Comparison of the angular positions of the main peaks in the sample highlights some displacement compared to the models of Treacy and Higgins [48], which proves that the sample analysed is not monomineral; that is to say, mordenite is spatially associated with other complex phases that alter the reflection of its peaks during analysis. Smectite (montmorillonite) shows the peak (pk.1) of maximum intensity (Imax: 100%) at the angular position of 2θ: 5.58, relatively masked by a mordenite peak. Another six peaks of medium intensity were detected at the following 2θ angular positions: pk.3 (57.88), pk.5 (19.0) and pk.4 (11.69). The lower intensity peaks, pk.2, pk.7 and pk.6, were located at the angular positions 2θ: 17.14, 54.05 and 40.30, respectively.

The reflections of quartz peaks are observed in several angular positions (2θ). The authors only highlighted peaks of greater intensity. The peak of highest intensity (Imax: 100%) was located at position 2θ: 26.54. Other peaks of medium intensity were located at the following 2θ positions: 20.76, 36.45, 39.36, 40.19, 42.35, 45.70, 50.05, 54.79 and 55.23.

Illite has its main peak at position 2θ: 8.81 with a relative intensity between 37 and 90%. Other secondary peaks are located at positions 2θ: 9.0, 26.88, 28.11, 35.62, 45.67 and 55.06, with intensities that vary between 1.56 and 41%.

Muscovite shows its main peaks at positions 2θ: 8.85, 19.67 and 34.89. The reflections of the secondary peaks were located at 2θ: 8.84, 19.72, 25.46, 35.13 and 53.05.

The main peaks of sodium plagioclase were located at 2θ: 26.58, 25.70 and 27.74, with Imax: 100%. Peaks of calcic plagioclase were seen in positions 2θ: 25.62, juxtaposed to the mordenite at 2θ: 28.03, with a relative intensity of 75%.

The minerals described in the analysed sample are especially important for the objectives of this research. Their origin is confirmed to be pyroclastic of zeolitic tuffs, as was established before by Stamatakis et al. [49]. It can be deduced that the fragments and crystals of quartz, plagioclase and volcanic glass, among others, were broken, pulverised and reheated during pyroclastic processes, increasing their surface area and the sintering of some components that brought about amorphous phases. Both factors give the analysed sample a strong pozzolanic reactivity, reinforced by the cation exchange capacity (CEC) and absorption-adsorption-desorption (AAD) properties of mordenite, smectite (montmorillonite) and halloysite [50,51].

**Figure 2:**
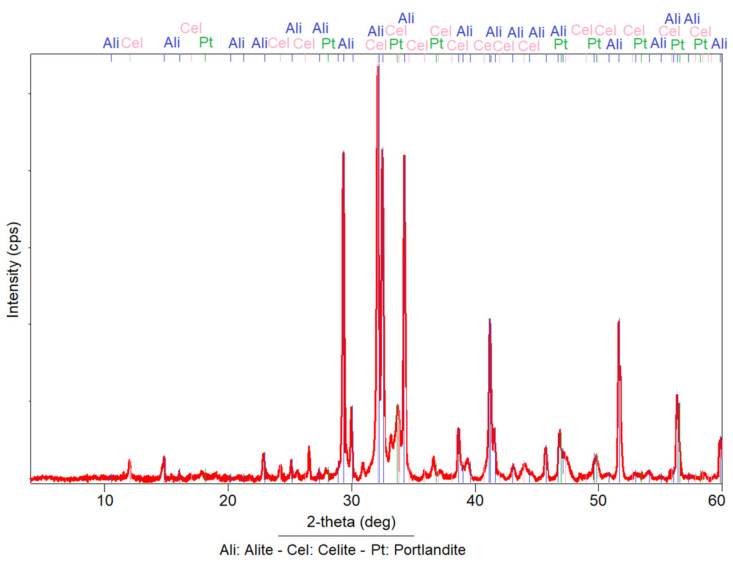
X-ray diffraction patterns of reference portland cement used in this work.

The analysed anhydrous cement sample produced different peaks characteristic of anhydrous crystalline species. The phases observed were composed of tricalcium aluminate (Alite), tetracalcium alumino-ferrite (Celite) and tricalcium silicate (Portlandite); being the alite the main mineral phase (Figure 2).

**Figure 3:**
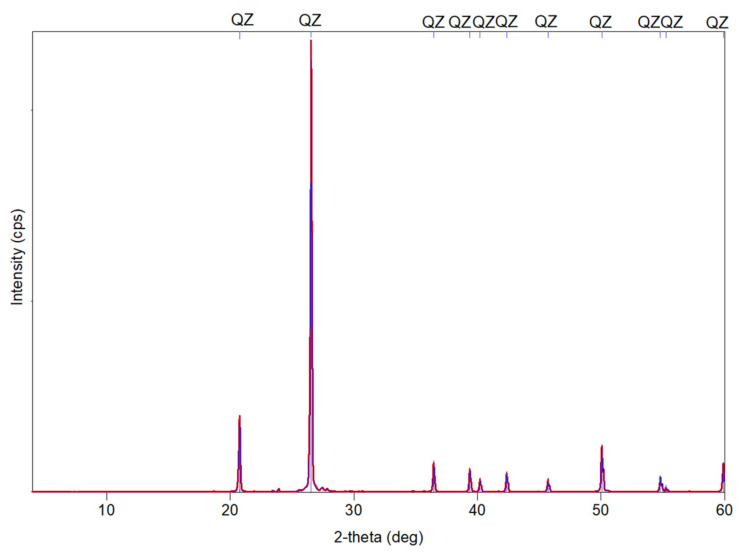
X-ray diffraction patterns of the standard sand used in this work.

Standard sand (SS) has two mineral phases, the main phase composed of quartz and a secondary phase of feldspar. According to UNE-EN 196-1:2018 [45], the quartz content of this sand is 98% (Figure 3). The study of the phases indicated that the SS is very pure, highly physically and chemically stable.

### 3.2. Scanning Electron Microscopy (SEM)

**Figure 4:**
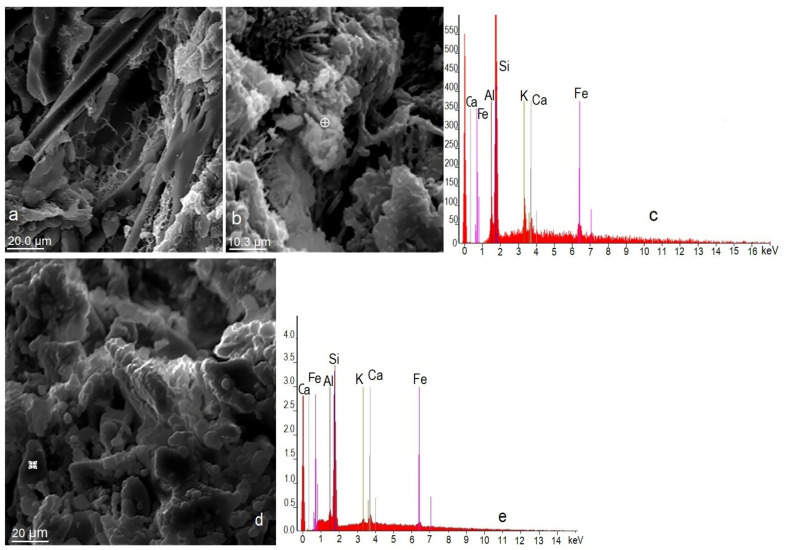
Natural mordenite microphotographs used in this research (**a**,**b**,**d**) and EDX diagrams (**c**,**e**).

The mordenite shows good crystalline development and exhibits varied and different morphologies (Figure 4a,b,d). The crystals are elongated, fibrous, acicular and tabular (Figure 4a,b). They form aggregates that are compact linear, parallel, dendriform and radial. Occasionally mordenite crystals form very compact crystalline aggregates, as shown in Figure 4d. The colour of the mordenite is white. The smectites are often intergrown with mordenite forming massive and sometimes radial aggregates, both of which have a genetic and spatial relationship that is seen in many parts of southern Spain; this aspect was pointed out by other authors [52,53]. Mordenite crystals are idiomorphic when their sides are geometric and regular; other times the crystals are partially geometric (hypidiomorphic) or are seen to have a totally irregular morphology (xenomorphic). The largest size of these more developed crystals exceeds 25 µm. Smaller crystals clearly grow at the expense of larger crystals.

The mordenite crystals grow at the expense of altered volcanic glass, as is seen in Figure 4a, which corroborates what was stated by Kitsopoulos [54], IZA Commission [55] and Costafreda [53]. According to the EDX diagrams in Figure 4c,e, the Fe content is too excessive for a chemical analysis of mordenite crystal, which can be attributed to mordenite/smectite intergrowth.

### 3.3. X-ray Fluorescence (XRF)

**Figure 5:**
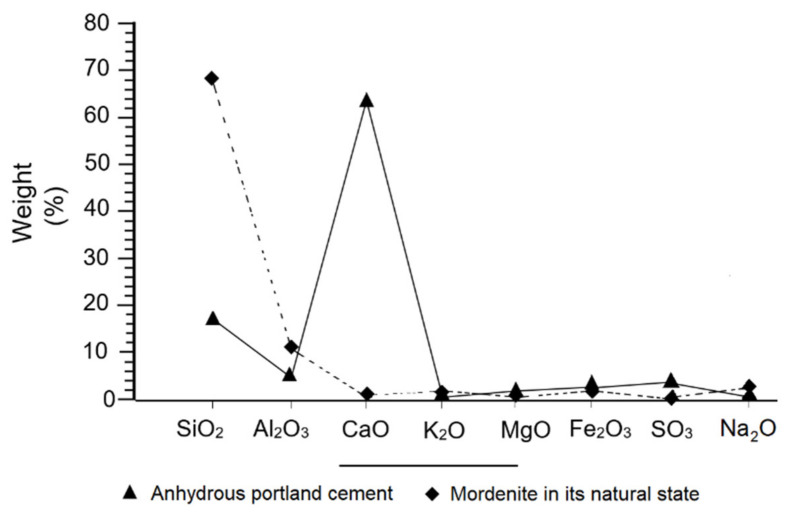
Behaviour of major compounds in the composition of natural mordenite and portland cement.

The chemical composition of the zeolite shows a high content of SiO_2_ and Al_2_O_3_ contrasting strongly with the content of CaO (Figure 5). In general, the contents of alkaline compounds (Na_2_O = 2.89% and K_2_O = 1.38%) stand out over the alkaline-earth (MgO = 1.29% and CaO = 1.15%) (Table 2). The presence of both compounds in the sample indicates the calc-alkaline character of the mordenite, predominantly alkaline.

In the case of portland cement, the high content of CaO (64.04%) regarding the SiO_2_ (17.45%), the Al_2_O_3_ (5.59%) and the SO_3_ (4%), point to a normal composition, within regular parameters (Figure 5) [56].

Figure 5 compares the composition of the portland cement to the researched mordenite (Mor), showing a noticeable difference between the contents of SiO_2_, Al_2_O_3_ and CaO, with an inverse type of ratio, where an increase in the silica and alumina content of the mordenite translates as a lower amount in the PC, on the contrary, an abnormal percentage of CaO in the PC is almost negligible in mordenite. This inverse ratio was also observed in the higher SO_3_ content of PC compared to the extremely low content of this compound in mordenite. Finally, this difference in loss on ignition values was found to be exceptionally high in mordenite (12.53) and extremely low for PC.

The composition of the SS is highly homogeneous and practically mono mineral, which indicates that SiO_2_ is the most abundant compound by far, followed by Al_2_O_3_. The presence of both compounds signals two fundamental mineralogical phases in the SS, consisting of quartz and feldspar. The virtually negligible presence of other major compounds, as well as the low loss on ignition values (LOI = 0.48%), indicate the high level of purity of this sand.

### 3.4. Granulometric Analysis

#### 3.4.1. Mordenite Granulometric Analysis: Fine Particle Diameter and BET Surface

**Figure 6:**
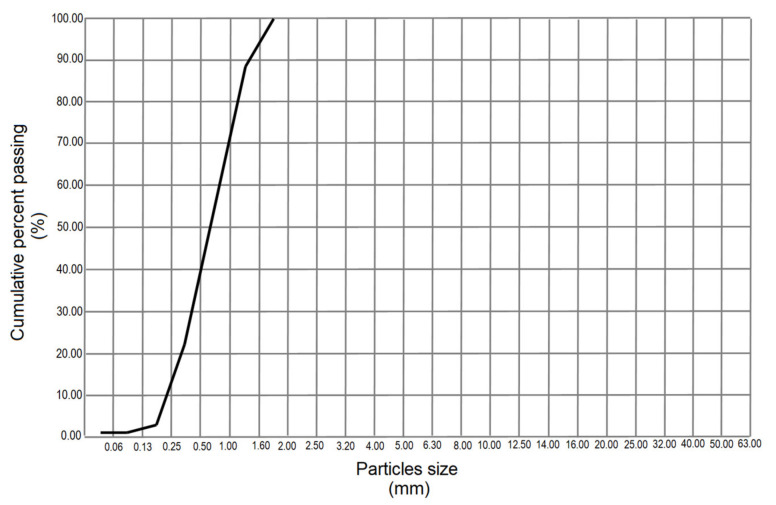
Granulometric curve of natural zeolite particles passed through the filler.

Fine and uniform granulometry was obtained, below 63 µm (Figure 6). This particle diameter was considered suitable to ensure hydraulic reactivity in the mortar and therefore the highest mechanical strength. In addition to this, hydration is most effective when the particle diameter is smaller [57].

The specific surface area (BET) on the investigated mordenite was 66 m^2^/g with an initial particle diameter of 2.88 μm and another final diameter equal to 139.25 μm. The average value was 54.76 μm which turned out to be quite close to that of Blaine granulometry.

**Figure 7:**
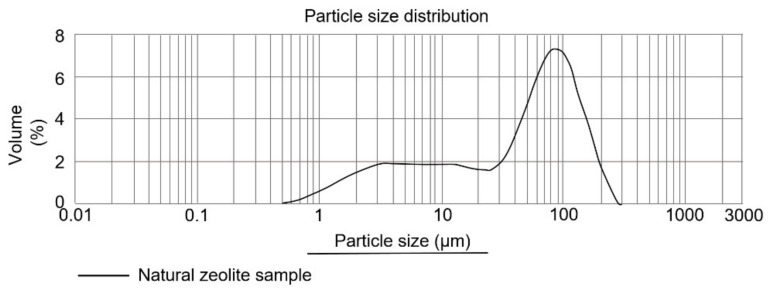
Particle size distribution vs. zeolite sample volume.

Figure 7 shows the regularity and uniformity of the grinding and sieving process of the mordenite sample. The average pore size value was 0.835 nm, much like what was calculated by Costafreda [53]. This is also confirmed by Payra and Dutta [58], who have established that the pore size in the mordenite varies between 0.2–0.850 nm.

#### 3.4.2. Granulometric Analysis of Standard Sand

**Figure 8:**
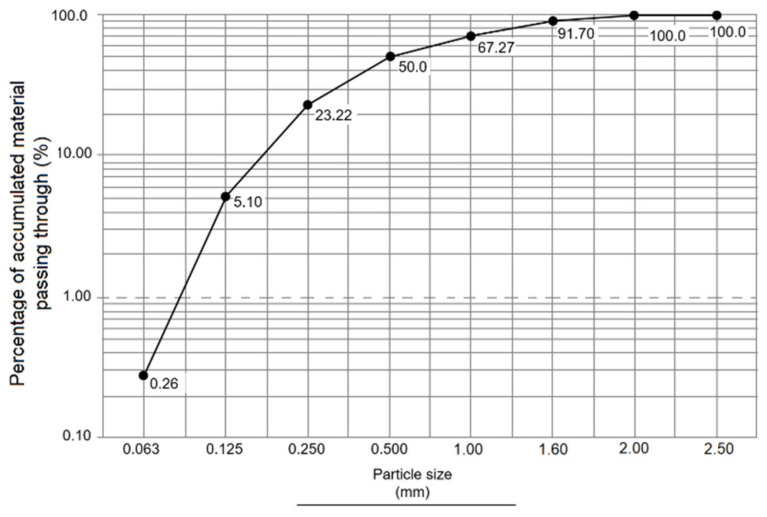
Standard sand granulometric curve.

Figure 8 shows a regular distribution of the standard sand (SS) particles. The granulometric fractions were in the range of 1.0 and 0.5 mm (24.48 and 32.43%, respectively), these being the ideal diameters to make mortar mixtures. Granulometric fractions that were lower than the above were discarded to prevent excess water absorption, while coarse ones with similar diameters were concentrated according to the Standard UNE-EN 933-1:2012 [42]. The coarse fragments ensure the gradual increase in the mechanical strength of the mortars, which permits all silica contained in the standard sand to react with the cement–water interface [53,59].

### 3.5. Effect of Mordenite on Initial and Final Setting Times of Fresh Mortar

**Figure 9:**
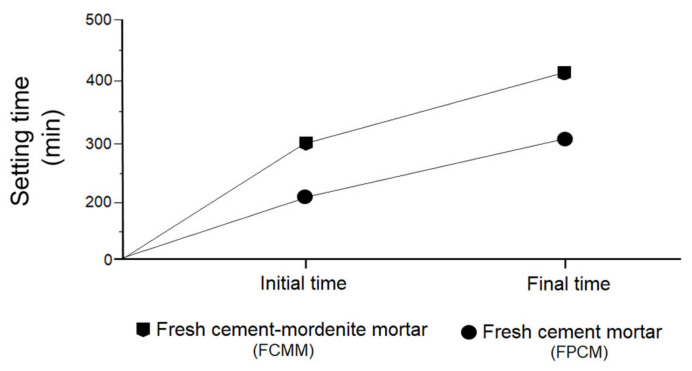
Initial and final times of settings.

A marked difference was detected in the initial and final times of fresh settings in both groups of mortars, both those made with cement/mordenite (FCMM) and portland cement (FPCM). In the FCMM the initial and final times of settings varied between 320–420 min, respectively, while for the FPCM it was 212–310 min, respectively (Figure 9). These results found that the effect produced on mortars was due to the pozzolanic character of mordenite which manifested as an alteration of the setting process in the early curing stages, as was mentioned before by Bilim [15].

The marked hydraulic properties of portland cement reduced setting times and caused the mortar to harden in a very short time, as some researchers have confirmed such as Kocak et al. [60]. In the FCMM, the increase in mechanical strength developed slowly, up to almost 28 days of age. In addition to this, the volume stability or expansivity was determined using the Le Chatelier apparatus, according to Standard UNE-EN 197-1:2011 [39]. The results indicated that the FCMM barely expanded (0.09 mm). The Standard UNE-EN 197-1:2011 [39] fixes the limit of this expansion at ≤10 mm.

### 3.6. Effect of Mordenite on The Consistency of Fresh Mortar

**Figure 10:**
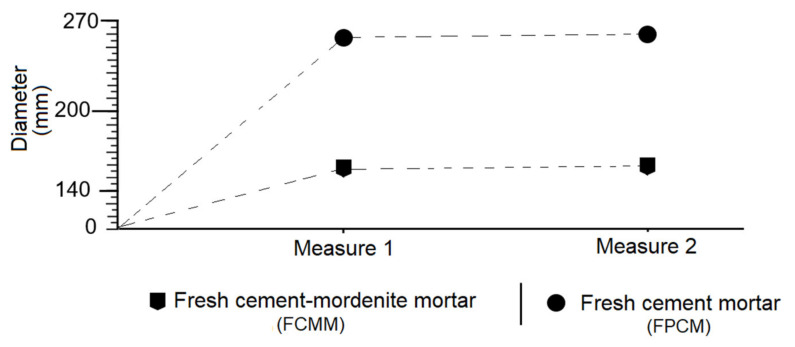
Consistency of fresh mortar of mordenite/cement (FCMM) and portland cement (FPCM).

The behaviour of the consistency was vastly different for both fresh mortar groups, the FCMM and the FPCM. In the first case, the diameter of the mortar mass expanded on the worktable was 154.5 mm, whereas for the second it was 260 mm, almost doubling the first one (Figure 10).

On the other hand, it was found that the presence of mordenite in the mixture caused a greater absorption of water, which was due to its degree of fineness, its nature as mesoporous solids and its cation exchange capacity (CEC). Some researchers such as Uzal and Turanh [61] have come to the same conclusion by researching with zeolites of the clinoptilolite and heulandite variety.

### 3.7. Effects of Mordenite on The Density of Fresh Mortar and Specimens

**Figure 11:**
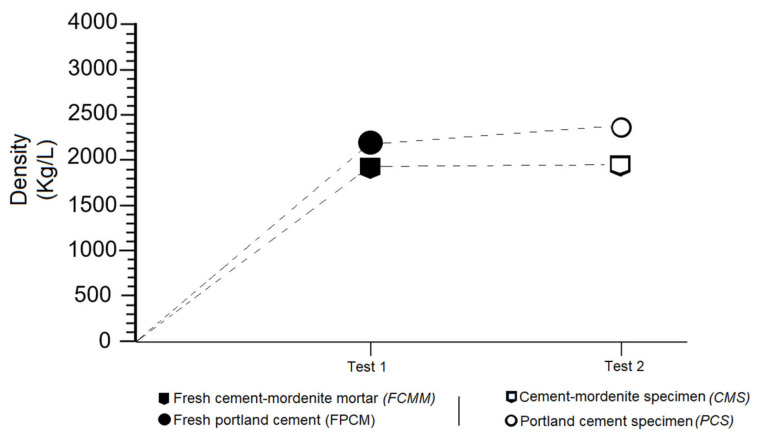
Density of fresh mortars (FCMM and FPCM) and specimens (CMS and PCS).

The results of this research demonstrated a low density of mortars with added mordenite, both fresh FCMM mortars and CMS specimens. In mortars containing only portland cement, a visibly higher density was determined, as can be seen in Figure 11. It was therefore established that mordenite can lighten the mortars, given their inherent low density.

**Figure 12:**
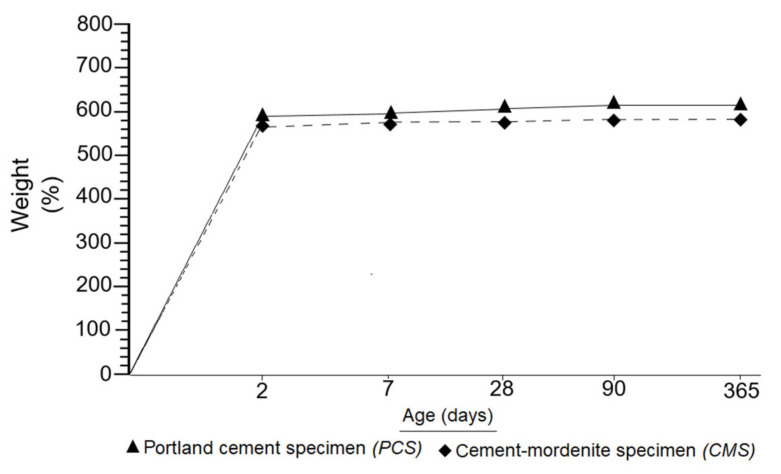
Comparison between the weight of CMS and PCS specimens.

Figure 12 confirmed the evidence set out in Figure 11. This research systematically weighed CMS and PCS, from 2 to 365 days of setting. Figure 12 shows that PCS are always heavier than CMS. Two key aspects were highlighted in this research: specimens with added mordenite are lighter and more resistant. Many researchers, including Karakurt et al. [62], Barnat-Hunek et al. [63], Pekgöz and Tekin [64], Sai Teja et al. [65] and Vijayan et al. [66], among others, consider zeolites to be high-performance light aggregates capable of decreasing the excessive density of concretes and mortars.

### 3.8. Effect of Mordenite on The Behaviour of Mechanical Strength of The Mortars

**Figure 13:**
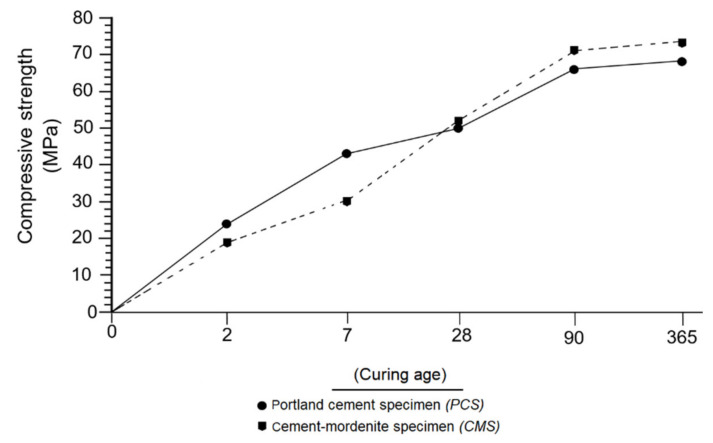
Evolution over time of the compressive strength of CMS and PCS.

Two types of mechanical strength were considered in the tests of the specimens: the initial one, consists of a period from 2 to 15 days, and the normal one, which starts after 28 days [45]. Figure 13 compares the values of compressive strength of the CMS and PCS specimens at different ages. Within two days of setting, the compressive strength of the PCS exceeded that of CMS, this fact became more evident at 7 days, which was due to the increased hydraulic reactivity of PC.

A different case occurred at 28 days, where the compressive strength of CMS exceeded the strength of PCS. This trend was still taking place at 90 and 365 days of curing, respectively. Similar to Janotka et al. [67] in their research with pozzolans, it was established in this research that the effect of mordenite as a pozzolan on mortars was present in the form of low compressive strength in short curing periods (2 and 7 days); however, at close to 28 days it was able to provoke a moderate increment in the compressive strength.

### 3.9. Effect of Mordenite on The Textural and Morphological Evolution of Minerals Formed during the Mortar Curing

**Figure 14:**
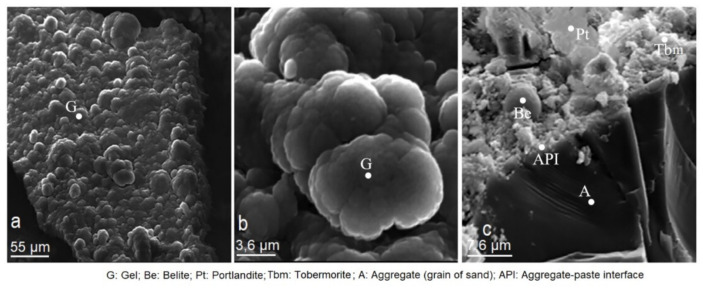
(**a**–**c**). Microphotographs of cement/mordenite specimens (CMS) taken at 7 days of age.

**Figure 15:**
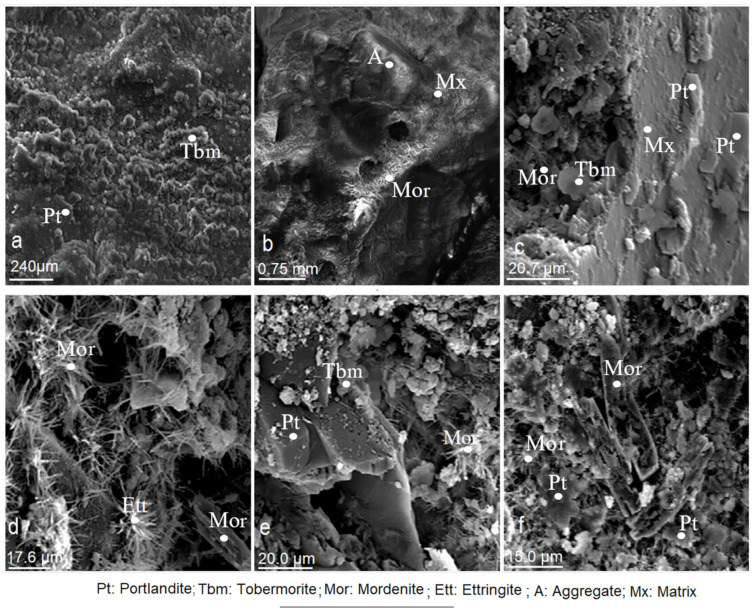
(**a**–**f**). Microphotographs of cement/mordenite (CMS) specimens taken at 28 days of age.

**Figure 16:**
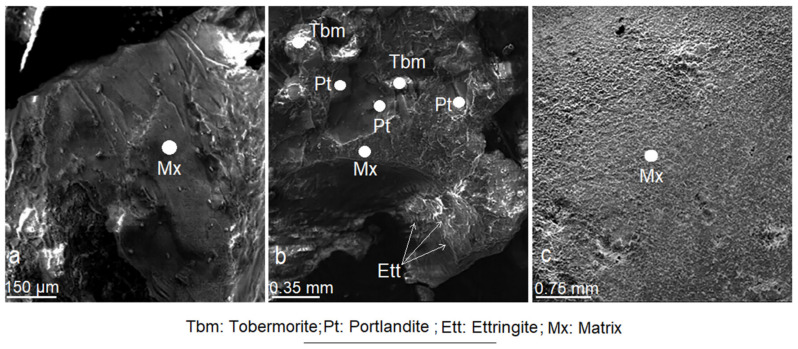
(**a**–**c**). Microphotographs of cement/mordenite specimens (CMS) taken at 90 days of age.

**Figure 17:**
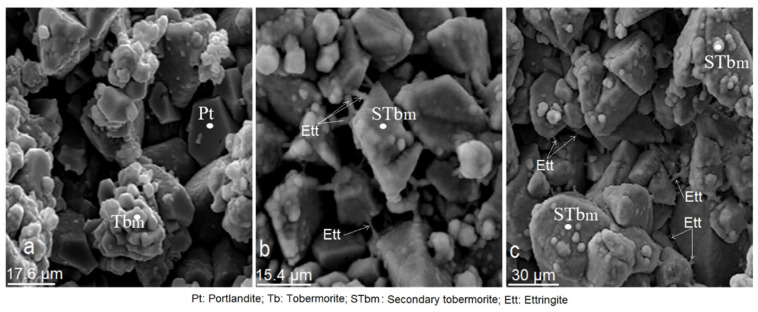
(**a**–**c**). Microphotographs of cement/mordenite specimens (CMS) taken after 365 days.

This research monitored the evolution of mineralogical properties that took place within the CMS at different ages (Figure 14, Figure 15, Figure 16 and Figure 17). At 7 days of age, no defined crystal nucleation had yet been formed. Reaction products consisted of tobermorite and portlandite gels that did not provide mechanical strength to mortar (Figure 14a,b). Exceptionally, some belite crystals were formed because of the arid-paste reaction (Figure 14c).

The evolution up to 28 days was even more evident (Figure 15a–f). The matrix became more compact, without pores, with a manifest blending of the arid-paste interfaces (Figure 15a,b). There was a good development of the mineralogical phases made of portlandite, tobermorite and ettringite (Figure 15c–f). The crystals have well-defined geometric shapes, as well as hexagonal, acicular and bacillary habits. The mordenite crystals are visible in Figure 15b–f. Occasionally, these crystals are not visible (Figure 14a–c, Figure 15a, Figure 16 and Figure 17), as they were masked from the beginning of the setting by the gels and subsequently by the crystallised tobermorite that adhered to the surface of the mordenite. This trend remained throughout the research period of the setting.

According to Figure 16a–c, at 90 days of setting, with the process of crystallisation and compaction, the specimens experienced a greater degree of cohesion, where one of the most characteristic features of the matrix was its virtually cryptocrystalline texture. In Figure 16b tobermorite, portlandite and ettringite crystals are observed in a consolidated matrix.

Analysis of CMS at 365 days of age showed a development attained by tobermorite and portlandite crystals in the form of compact crystalline aggregates. Ettringite crystals have not undergone abnormal growth, and generally appear connected to portlandite crystals (Figure 17b,c).

Portlandite forms hexagonal and sometimes trigonal plates and exhibits a marked idiomorphism (Figure 17a). The tobermorite forms massive cumulus and intercumulus granular crystallines, which are strongly bonded to each other. Its crystals are shaped like rosettes, fibres, compact masses or cylinders that connect the portlandite crystals (Figure 17b,c). The tobermorite always appears attached to the portlandite. Generally, the tobermorite forms layers that cover the surface of the portlandite crystals, due to a substitution process with secondary tobermorite formation (Figure 17b,c) [68]. The effect of mordenite on CMS was interpreted as a rapid reaction with portlandite, which favoured primary tobermorite formation. The pozzolanic capacity of the mordenite slowed the hydration process of calcium aluminates, allowing dicalcium silicates to react and produce more tobermorite. Subsequently, secondary tobermorite was formed by the replacement of the portlandite (Figure 17a–c). This coincides with what is stated by Costafreda [53].

### 3.10. Effects of Mordenite on The Growth of Minerals Formed during Mortar Curing

**Figure 18:**
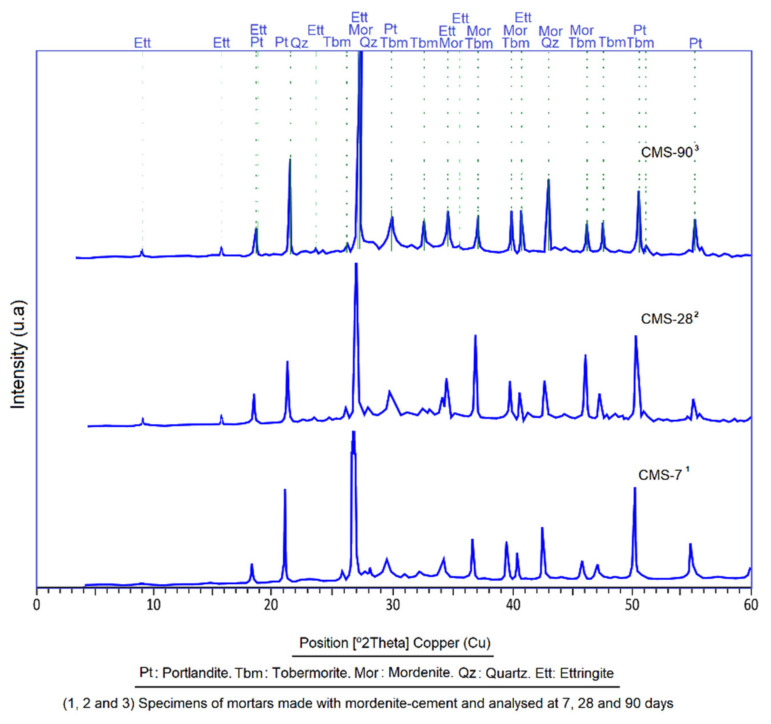
X-ray diffraction patterns showing the evolution in time of the mineral phases found in CMS.

This analysis showed the development and evolution of the mineral phases during the progressive curing of CMS (Figure 18) and confirmed what was described in the previous section. X-ray diffraction patterns reflect the effect of mordenite through the intensity of the main peaks, which are increasingly intense, as the age of the researched specimens increases.

The first peaks appear at 7 days of age, due to the reaction of the main products such as portlandite, tobermorite and small traces of ettringite. The appearance of the most intense peaks of quartz is due to an increase in silica from the mordenite and standard sand (SS) (Figure 18). The fundamental changes were the attenuation of the main peaks of mordenite as a result of its reaction with Ca(OH)_2_; important reflections also occurred on portlandite peaks. At this stage, the tobermorite produced rather small, low development peaks, testimony to a slow rate in coagulation and crystallisation of gels. The silica may also have formed from the appearance of silicate phases of secondary origin, with strong crystalline character.

At 28 days of age, the growth of the tobermorite peaks was increased, which indicated a process of hydration of the alite and belite with the consequent crystallisation in the form of tobermorite; at the same time, attenuation occurred at the peak of the portlandite which was interpreted as a secondary tobermorite substitution process. At this age, ettringite peaks are visible as well (Figure 18).

At 90 days there was a marked increase in the intensity of reflections of the main peaks of the tobermorite and the portlandite, indicating a maximum degree of crystallisation of these minerals; however, ettringite peaks do not show significant development (Figure 18).

### 3.11. Effects of Mordenite in the Chemical Composition of Mortars

**Figure 19:**
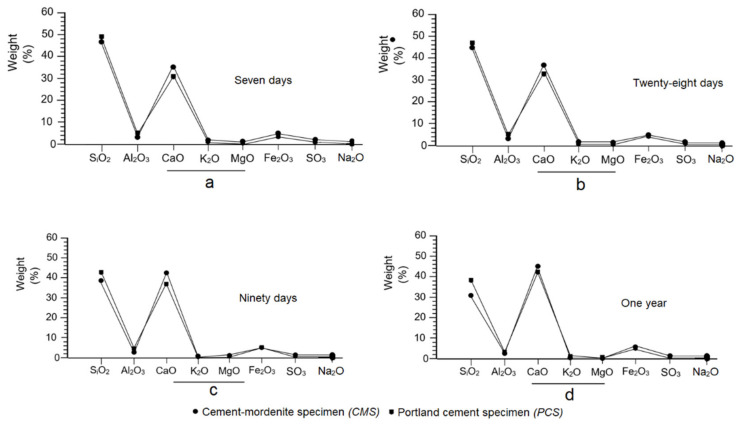
Behaviour of major compounds in cement/mordenite (CMS) and portland cement (PCS) specimens at different ages: (**a**) at 7 days, (**b**) at 28 days, (**c**) at 90 days and (**d**) 1 year.

In the curing process, there were obvious changes in the chemical composition of CMS. Initially, the chemical composition of natural mordenite was compared with that of anhydrous portland cement, as seen in Figure 5, and a big difference was observed in the contents of SiO_2_, AI_2_O_3_, and CaO; whereas in the K_2_O, MgO, Fe_2_O_3_, Na_2_O and SO_3_ compounds, the difference was not as great, as can be seen in Figure 19a–d. That is to say, the difference in the percentages of SiO_2_, Al_2_O_3_ and CaO tended to be smaller in the CMS, as shown in the diagrams in Figure 19 at different ages.

Figure 19a–d shows that the increase in CaO over time was more pronounced in the PCS than in CMS. In the latter there was a gradual fall in SiO_2_ and the Al_2_O_3_ between 7 and 365 days, and a noticeable and gradual increase in CaO over the same period; this was because the hydraulic reaction system consumed a lot of SiO_2_ and Al_2_O_3_ to neutralise the excess alkalinity in the paste, as well as for the transformation of the portlandite into secondary tobermorite, consequently gaining mechanical strength.

Moreover, the nucleation and crystallisation of the tobermorite required a substantial amount of SiO_2_, which caused a deficit of this compound [56]. Another aspect to note is the contribution of mordenite to the decrease in SO_3_ in the CMS at 28, 90 and 365 days of curing. However, there is a negligible difference between the SO_3_ contents found in CMS and PCS (Figure 19a–d).

According to the evolution shown in the diagrams in Figure 19a–d, the SiO_2_:CaO ratio is inverted; meaning, that in the reaction system it took more SiO_2_ to fix more lime. On the other hand, K_2_O and Na_2_O in the CMS decreased between 7 and 365 days, which was because of the cationic exchange capacity (CEC) of the mordenite during the hydraulic reaction process. The researched mordenite has high contents of Al_2_O_3_ (Figure 5), which inhibited the instant reaction of C_3_A and the massive formation of ettringite, as explained above [69]; at the same time, it favoured the slow and complete hydration of the belite and tobermorite, and positively influenced the increase in mechanical strength in the CMS, as Borštnar et al. [70] have already noted.

## 4. Conclusions

The conclusions of this research are detailed in the following lines:

1. The mordenite sample analysed in this research has a complex mineralogical composition where different mineral phases coexist. Mordenite is the main mineralogical phase and is accompanied by secondary phases such as smectite (montmorillonite), halloysite, illite, muscovite, biotite, plagioclase, quartz and amorphous phases.
2. The chemical composition of the samples is calc-alkaline, where the major compounds SiO_2_, AI_2_O_3_, K_2_O and Na_2_O, stand out, as well as a high loss on ignition.
3. Mortars made with partial substitution of cement with mordenite (CMM) showed the following properties regarding portland cement mortars (PCM): longer initial and final setting times, greater consistency, lower fluidity, lower density, lower weight and greater mechanical strength from 28 to 365 days.
4. The study of CMS by DRX and SEM showed that the presence of mordenite slows down the hydration process, as seen in the longer initial and final setting times, which regulate the rapid hydration of C_3_A and allows slow-reacting silicates to be completely hydrated. As a result, a large amount of primary and late tobermorite has formed (28, 90 and 365 days), resulting in the formation of secondary tobermorite at the expense of portlandite. Mordenite´s action has made the ettringite formation a process that is not anomalous.
5. According to what was analysed and discussed above, it is evident that the properties of mordenite, such as solid porous, cation exchange capacity (CEC), absorption-adsorption-desorption (ADD) properties, high contents of SiO_2_, Al_2_O_3_, K_2_O and Na_2_O, high LOI values, the presence of accompanying amorphous phases, as well as the long initial and final setting times and high mechanical strength deeply influence the positive evolution of the mortar setting, as was stated in the objectives and discussion of this work.
6. In addition, the results achieved proved that mordenite behaves like a quality natural pozzolan, capable of partially replacing portland cement without affecting mechanical strength. On the other hand, the properties described justify their use in the manufacture of pozzolanic cements and mortars that are lighter, more resistant to overly aggressive natural environments, and more environmentally friendly, mainly in relation to low CO_2_ emissions.
7. Finally, this work is new as it highlights the only deposit of zeolite of a mordenite type known in Spain.

According to the conclusions, the objectives of this research were fulfilled, thus, both the methodology used and the results obtained could be used in the integral characterisation of any pozzolanic material and in the manufacture of mortars, concretes and pozzolanic cements.

## Figures and Tables

**Table 1:** Proportions of materials in CMM and PCM mortars and their test ages.

Specimen Sample	Composition	Proportion (g)	Test Age (Days)
CMS-7 * CMS-28 CMS-90 CMS-365	Cement Mordenite Standard sand Distilled water	Cement (375) Mordenite (125) Distilled water (225) Standard sand (1350)	7/28/90/365
PCS-7 ** PCS-28 PCS-90 PCS-365	Cement Standard sand Distilled water	Cement (450) Distilled water (225) Standard sand (1350)	7/28/90/365

* CMS-7 to 365: Specimens made with cement/mordenite for testing at 7, 28, 90 and 365 days. ** PCS-7 to 365: Specimens made of portland cement for testing at 7, 28, 90 and 365 days (reference specimens).

**Table 2:** Chemical composition (% weight) of the natural mordenite sample used in this research, and determined by XRF.

Sample	S_i_O_2_	Al_2_O_3_	CaO	Na_2_0	K_2_O	MgO	Fe_2_O_3_	SO_3_	LOI	Si/Al
Mordenite	68.30	11.95	1.15	2.89	1.38	1.29	1.56	0.03	12.53	5

## Data Availability

Not applicable.
